# Morphological and Functional Changes in the Retina after Chronic Oxygen-Induced Retinopathy

**DOI:** 10.1371/journal.pone.0032167

**Published:** 2012-02-14

**Authors:** Shinsuke Nakamura, Shunsuke Imai, Hiromi Ogishima, Kazuhiro Tsuruma, Masamitsu Shimazawa, Hideaki Hara

**Affiliations:** Molecular Pharmacology, Department of Biofunctional Evaluation, Gifu Pharmaceutical University, Gifu, Japan; Dalhousie University, Canada

## Abstract

The mouse model of oxygen-induced retinopathy (OIR) has been widely used for studies of retinopathy of prematurity (ROP). This disorder, characterized by abnormal vascularization of the retina, tends to occur in low birth weight neonates after exposure to high supplemental oxygen. Currently, the incidence of ROP is increasing because of increased survival of these infants due to medical progress. However, little is known about changes in the chronic phase after ROP. Therefore, in this study, we examined morphological and functional changes in the retina using a chronic OIR model. Both the a- and b-waves in the OIR model recovered in a time-dependent manner at 4 weeks (w), 6 w, and 8 w, but the oscillatory potential (OP) amplitudes remained depressed following a return to normoxic conditions. Furthermore, decrease in the thicknesses of the inner plexiform layer (IPL) and inner nuclear layer (INL) at postnatal day (P) 17, 4 w, and 8 w and hyperpermeability of blood vessels were observed in conjunction with the decrease in the expression of claudin-5 and occludin at 8 w. The chronic OIR model revealed the following: (1) a decrease in OP amplitudes, (2) morphological abnormalities in the retinal cells (limited to the IPL and INL) and blood vessels, and (3) an increase in retinal vascular permeability via the impairment of the tight junction proteins. These findings suggest that the experimental animal model used in this study is suitable for elucidating the pathogenesis of ROP and may lead to the development of potential therapeutic agents for ROP treatment.

## Introduction

Retinopathy of prematurity (ROP) is a disease that affects the retinas of premature infants. The key pathological change, namely, retinal neovascularization, is associated with local ischemia followed by the subsequent development of neovascularization. In the more severe forms of the disease, the abnormal vascular changes may progress to retinal detachment. Once retinal detachment occurs, the prognosis for recovery of good visual acuity is poor. As a result, ROP is the leading cause of preventable childhood retinal dysfunction. The World Health Organization's Vision 2020 programme targets ROP as an avoidable disease requiring early detection and treatment to prevent blindness [1].

The severity of ROP is graded in stages from 1 to 5. Blindness from ROP is caused by retinal detachment due to tractional retinal angiogenesis (stage 5). Recently, it was reported that even mild ROP (stages 1 or 2) can cause permanent functional sequelae [2], [3]. Rod-mediated abnormalities are involved in the regulation of eye growth in children with mild ROP [2]. However, little is known about the causes of the blindness that occurs in the absence of retinal detachment. Further research is required to elucidate the underlying causes of ROP, clarify the chronic functional and morphological changes, and propose new prevention strategies.

The oxygen-induced retinopathy (OIR) model is widely used for studies of retinal neovascular diseases such as ROP and diabetic retinopathy [4], [5]. The OIR model mirrors events that occur during ROP, including the pathological alterations that affect premature infants. At postnatal day (P) 18, ROP model rats show significant abnormalities in both the retinal vasculature and neural function [6]. Dysfunction of the neural retina, including photoreceptors and post-receptors, has been documented in patients with ROP and in the rat model [3], [7], [8], [9]. However, it is unclear why retinal function fails to return to normal even after the disappearance of abnormal neovascular tufts.

The blood-retinal barrier (BRB) is a selective barrier of the eye and is composed of capillary blood vessels. The BRB plays an essential role in protecting the neural tissues from toxic materials and maintaining the visual and neural functions of the retina. BRB breakdown is one of the most important pathophysiological changes in ischemic retinal diseases such as ROP and diabetic retinopathy [10], [11], [12]. In fact, retinal vascular permeability is higher in the rat OIR model than in normal rats; this occurs as a result of increased production of vascular endothelial growth factor (VEGF) [13]. Furthermore, degeneration of astrocytes has been reported to be involved in the failure of the BRB in the feline OIR model [14]. In developing mouse retinas, astrocytes trigger postnatal onset of radial vascular extension from the optic disc by secreting VEGF and depositing extracellular matrix scaffolds to which the migrating endothelial cells adhere [15], [16]. The barrier properties of blood vessels are attributed mainly to the presence of complex tight junction networks between endothelial cells [17]. Integral membrane proteins such as occludin [18], claudin [19], [20], ZO-1 [21], and junctional adhesion molecule (JAM) [22] are localized at tight junctions.

To create an effective therapeutic strategy for avoiding the blindness caused by ROP, it is essential to understand the relationship between retinal dysfunction and structure degeneration. Although there is evidence that abnormalities occur in both the retinal blood vessels and retinal cells in ROP [7], [13], [23], it is unknown how neuronal and vascular abnormalities are chronically related.

The purpose of the present study is to elucidate the interaction of the retinal blood vessels and retinal function using a chronic OIR mouse model.

## Results

### Visual dysfunction

**Figure 1:**
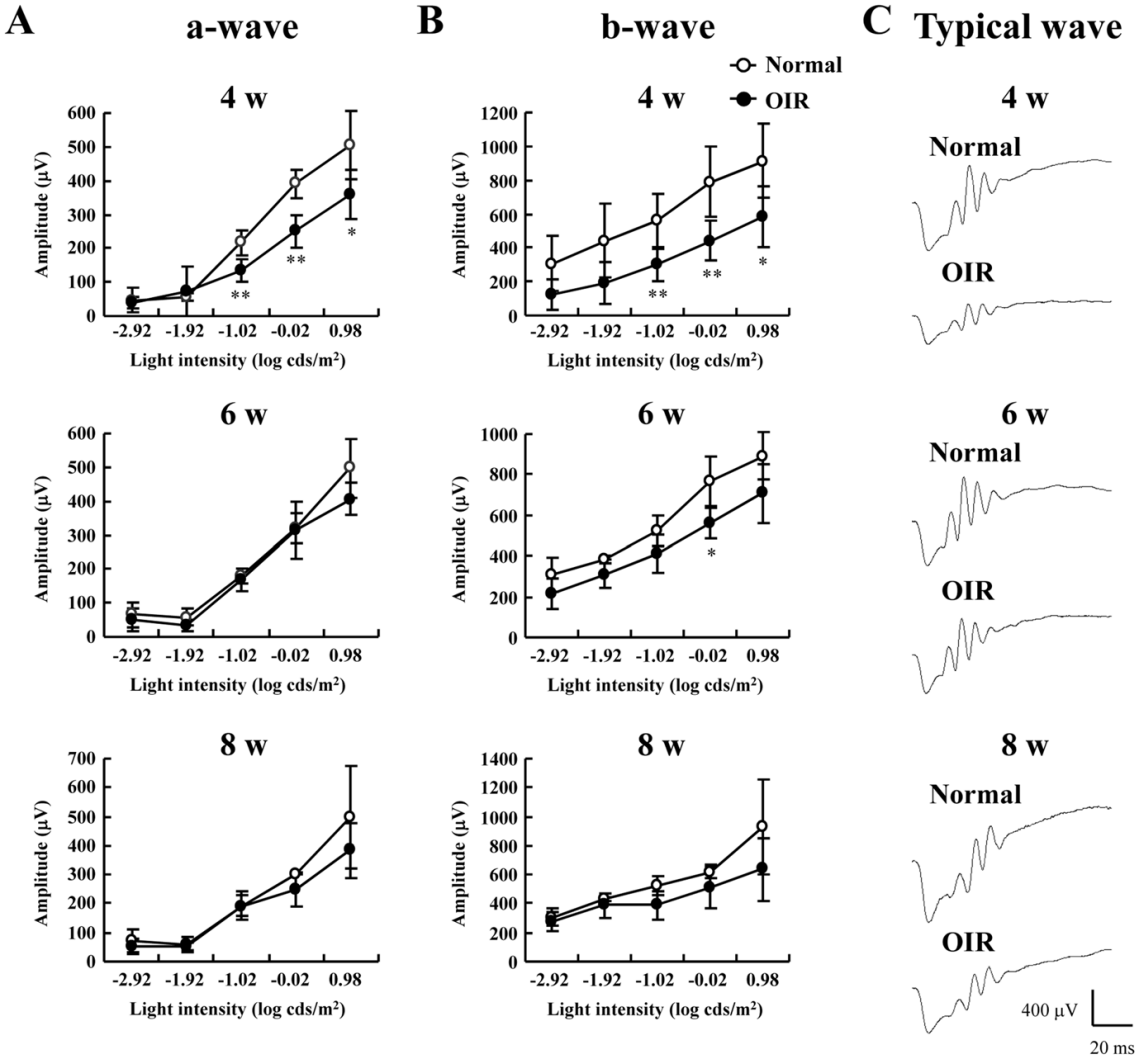
Measurement of dark-adapted electroretinography (ERG) amplitudes in the oxygen-induced retinopathy (OIR) model and normal mice. Amplitudes of a- (A) and b-waves (B) from the OIR model or from normal mice were measured at 4, 6, and 8 w. Stimulus flashes were used from −2.92 to 0.98 log cds/m^2^. (C) Representative ERG waveforms at 4, 6, and 8 w. Values are expressed as the mean ± S.D., n = 4 to 6. ^*^ *P*<0.05, ^**^ *P*<0.01 versus Normal. OIR, oxygen-induced retinopathy model.

**Figure 2:**
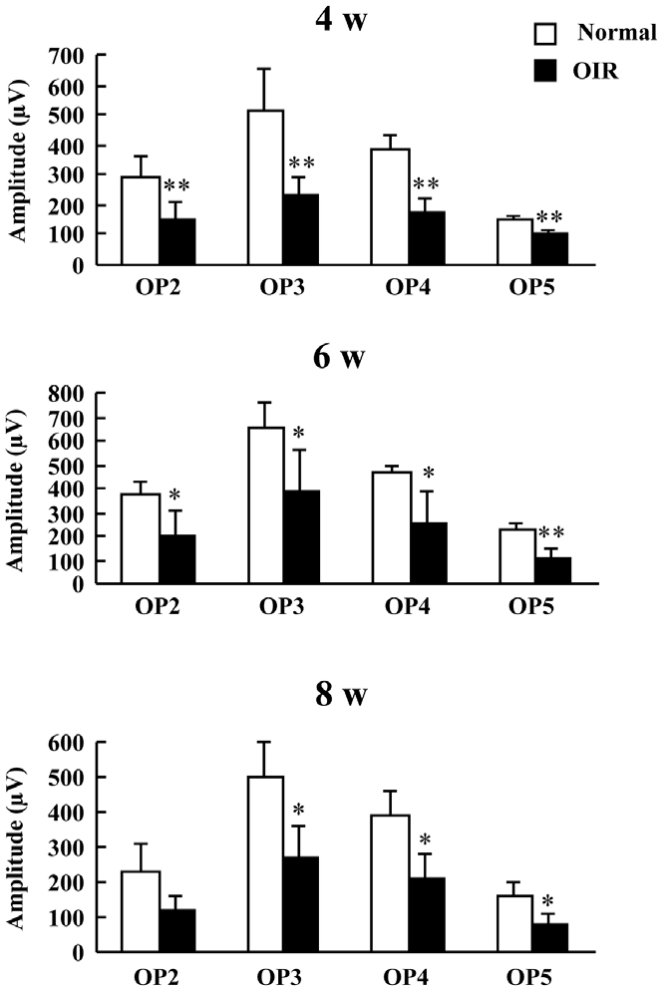
The oscillatory potentials (OPs) amplitudes in response to a light flash in the oxygen-induced retinopathy (OIR) model and normal mice. The averaged OP amplitudes were measured at 4, 6, and 8 w. Values are expressed as the mean ± S.D., n = 4 to 6. ^*^ *P*<0.05, ^**^ *P*<0.01 versus Normal. OIR, oxygen-induced retinopathy model.

To determine visual function after exposure to high oxygen, we performed ERG analyses. Similar to previous reports [24], [25], [26], OP1 was not analyzed because OP1 was contaminated by the a-wave. The peak-to-trough amplitudes of the individual OP wavelets (OP2, OP3, OP4, and OP5) were measured individually from stimulus onset to peak. To examine retinal function, we measured the amplitudes of the a- and b-waves. Both a- and b-wave amplitudes were significantly decreased in the OIR model mice at 4 w (Figs. 1A–C). However, in OIR model mice, we observed a time-dependent recovery of the decrease in the a- and b-wave amplitudes (Fig. 1–C). The OP amplitudes measured under scotopic conditions in response to a flash are shown in figure 2. For all OPs except OP2 at 8 w, the amplitudes for the OIR model mice at 4, 6, and 8 w were approximately 50% less than those in normal mice (Fig. 2).

### Retinal damage

**Figure 3:**
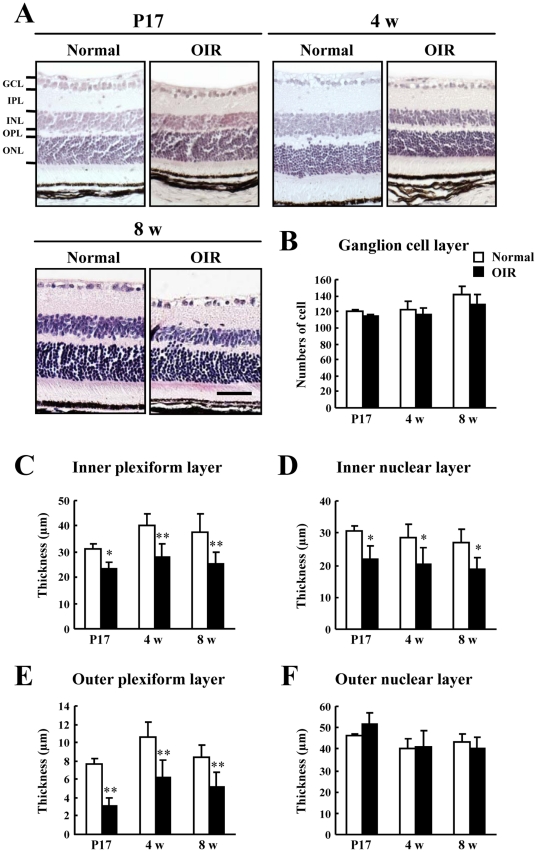
Retinal damage in the oxygen-induced retinopathy (OIR) model and normal mice. Retinal cross sections were prepared at 4 w and 8 w. (A) Hematoxylin and eosin staining. Scale bar, 50 µm. Retinal damage was evaluated by counting the number of cells in the GCL (B) and measuring the thickness of the IPL (C), INL (D), and ONL (E) in mice at 4 w and 8 w. Values are expressed as the mean ± S.D., n = 5 or 6. ^*^ *P*<0.05, ^**^ *P*<0.01 versus Normal. GCL, ganglion cell layer; IPL, inner plexiform layer; INL, inner nuclear layer; ONL, outer nuclear layer; OIR, oxygen-induced retinopathy model.

To investigate the damage in each retinal layer after exposure to high levels of oxygen during infancy, we evaluated the number of cells in the the ganglion cell layer (GCL) and the thicknesses of the inner plexiform layer (IPL), inner nuclear layer (INL), outer plexiform layer (OPL), and outer nuclear layer (ONL). In the OIR mouse model retinas, the thicknesses of the IPL, OPL, and INL decreased compared to those in normal mouse retinas at 4 and 8 w (Figs. 3A, C–E). However, there were no significant differences in the number of cells in the GCL and the thickness of the ONL between the OIR model and normal mice (Figs. 3A, B, F).

### Morphology of retinal blood vessels

**Figure 4:**
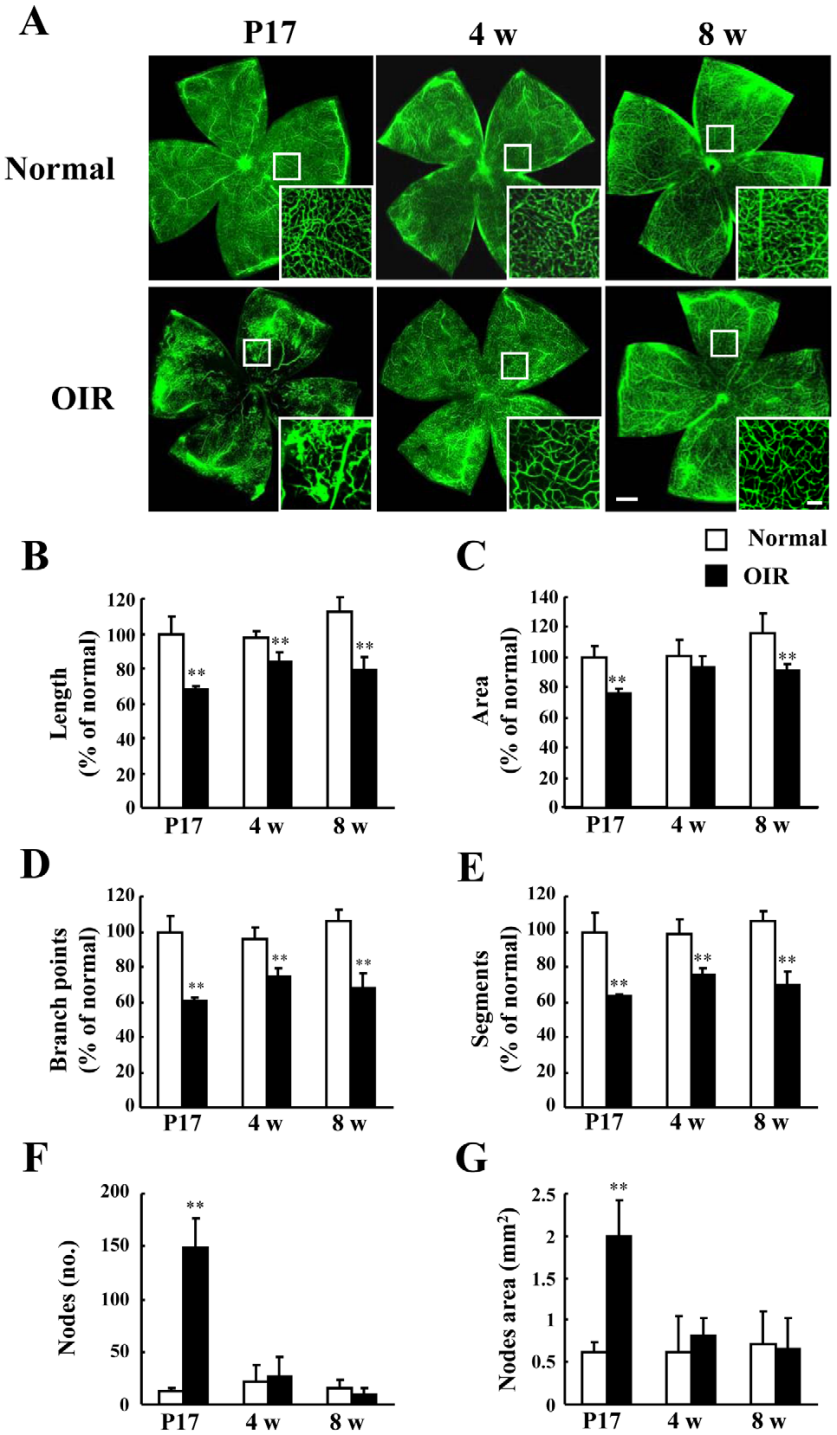
The development of retinal vascular lumen in the oxygen-induced retinopathy (OIR) model and normal mice. (A) Flat-mounted retinas in the OIR model mice and normal mice, along with (insets) enlargements, at 3 time points (P17, 4 w, and 8 w). Scale bars, 500 µm (100 µm in insets). Quantitative analysis of retinal vascular lumen at 4 w and 8 w was performed using an imaging analyzer (the Angiogenesis Tube Formation module) on the entire retina; 4 parameters were measured: (B) length, (C) area, (D) branch points, and (E) segments. Values are expressed as the mean ± S.D., n = 4 or 5. ^**^ *P*<0.01 versus normal. OIR, oxygen-induced retinopathy model.

We also examined retinal blood vessel morphology in OIR mice. As shown in Figure 4, retinal flat-mounts visualized by fluorescence angiography revealed significant abnormalities in the retinal blood vessels of OIR mice. To quantify retinal vascular development, we evaluated 4 parameters of retinal blood vessel formation using imaging software: length, area, branch points, and segments. At P17, abnormal vascular growth occurred in the OIR model mice but not in normal mice (Fig. 4A). Moreover, we found that the formation of retinal microvasculature significantly decreased with respect to length, branch points, and segment parameters in OIR model mice at P17, 4 w, and 8 w (Figs. 4B, D, E). Vessel area showed a decreasing tendency at 4 w, and we detected significant differences at P17 and 8 w (Fig. 4C). Furthermore, we investigated the change in retinal neovascularization between the OIR model and normal mice. As previously reported [27], the retinal neovascular tufts in the OIR model mice eventually disappeared after peaking at P17 (Figs. 4F, G).

### Permeability of retinal blood vessels

**Figure 5:**
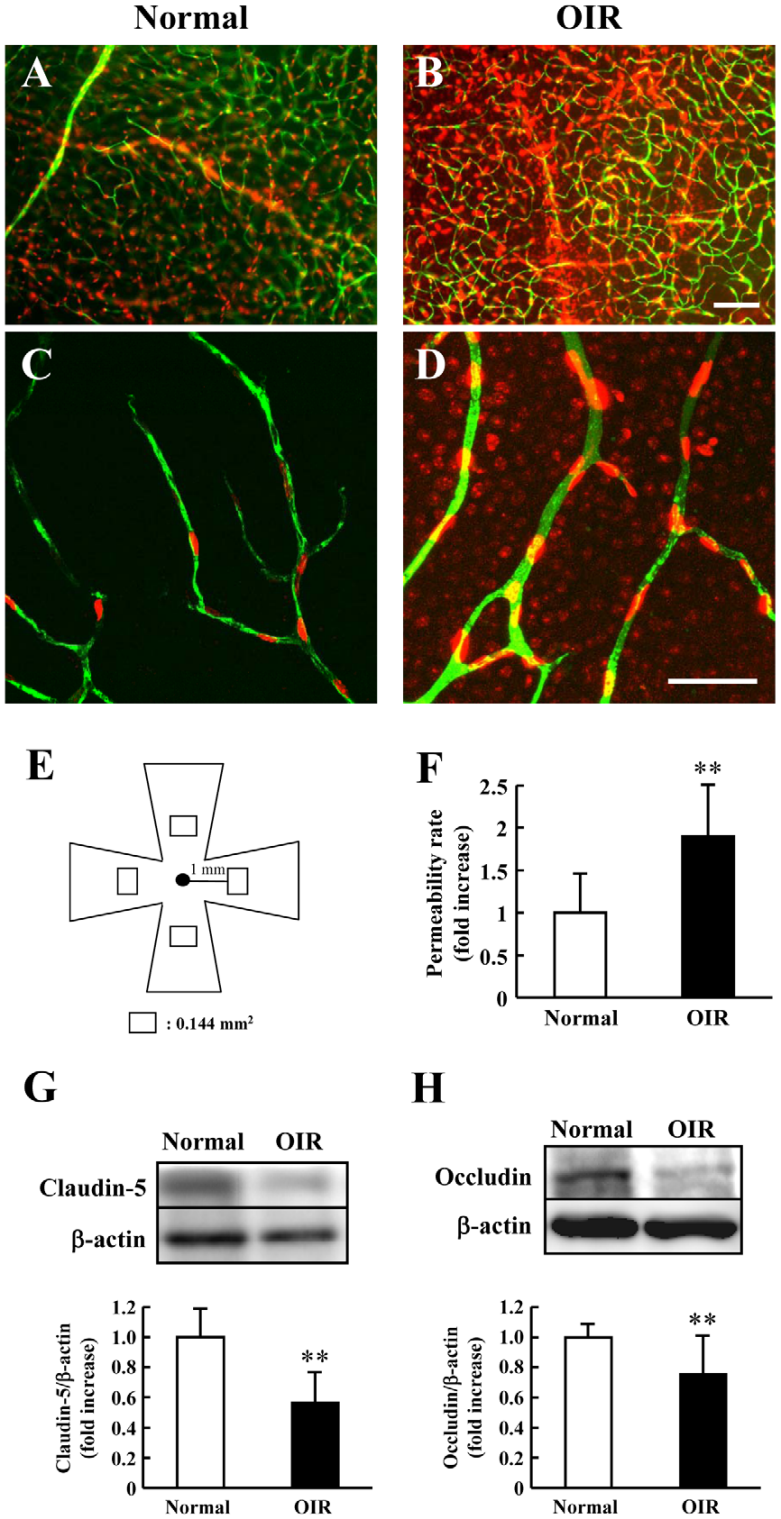
Retinal permeability in the oxygen-induced retinopathy (OIR) model and normal mice. Each upper image is shown using Metamorph (A, B). Confocal fluorescence micrographs (C, D) show a higher magnification version of part of the corresponding upper image. Scale bars, 100 µm (B) and 50 µm (D). Retinal permeability was evaluated in the 4 areas shown in (E) (each area 0.144 mm^2^×4 areas; total area 0.576 mm^2^) at 8 w. (F) Retinal permeability rate was quantified using mathematical formulae; see Materials and Methods for further details. Western immunoblots of claudin-5 (G), occludin (H), and β-actin proteins in the retina at 8 w in the OIR model mice and in normal mice. Expression was quantified by densitometry and corrected by reference to β-actin. Values are expressed as the mean ± S.D., n = 7 or 8. ^**^ *P*<0.01 versus normal.

The permeability of retinal blood vessels was evaluated by tracer experiments using FITC-dextran (2000 kDa) conjugated with Hoechst 33342 (616 Da). This tracer has previously been used to successfully evaluate the permeability of tight junctions of epithelial cells [20], [28], [29]. Injected FITC-dextran was detected in the vascular lumen. In addition, extravasation of Hoechst 33342 dye appeared as nuclear staining of surrounding retinal glial and neural cells, and the degree of staining was enhanced in the OIR model mice at 8 w (chronic phase) compared to that in normal mice (Figs. 5A–D). Quantitative analysis showed that the retinal permeability rate was significantly greater in the OIR model mice at 8 w (Fig. 5F). Moreover, western blot analysis confirmed significant decreases in both claudin-5 and occludin expression in the retina of the OIR model mice (Figs. 5G, H).

## Discussion

The main results of the present ROP study can be summarized as follows: (1) there were deficits in OPs, which are useful indices of IPL dysfunction; (2) retinal damage was found primarily in both the IPL and INL where many retinal blood vessels exist; (3) retinal vascular permeability increased via breakdown of the barrier properties in retinal blood vessels. These findings indicate that retinal hyperpermeability can cause retinal dysfunction and that the murine chronic OIR model is suitable for evaluating the pathogenesis of BRB disruption. Furthermore, these results demonstrate that the present model is useful as a chronic OIR model to clarify the relationship between visual function and retinal vasculature.

In this study, ERG readings reflected the loss of retinal function. To assess the retinal function in normal and OIR mice, the amplitudes of a- and b-waves were measured as they are indicative of photoreceptor function and the functional integrity of the INL, respectively [30], [31], [32], [33], [34], [35]. The cellular origin of OPs is thought to be primarily amacrine cells, although ganglion and bipolar cells may contribute to some parts of the OPs [35], [36], [37], [38]. In fact, OPs are helpful for identifying the progression of diabetic retinopathy. In the present study, there was a significant decrease in the amplitude of both a- and b-waves in the OIR mice compared to those in normal mice at 4 w (Figs. 1A, B). However, at 8 w (the chronic phase), there were no alterations in the amplitudes of either the a- or b-waves (Figs. 1A, B). Thus, transmission from photoreceptors may be impaired in the chronic OIR model. On the other hand, the present results revealed a uniform reduction in all OPs (Fig. 2). It has been reported that both a- and b-wave amplitudes were significantly decreased in the OIR rat at P30 and P60 [7]. Moreover, a previous study reported that the OP amplitudes were smaller in the OIR rat than in the normal rat at P67 [6]. In the chronic phase, although the recovery of both a- and b-wave amplitudes in the OIR mice was different from that in the rat OIR model, the decrease in the OP-wave amplitudes in the murine OIR model was in agreement with previous findings in the rat OIR model. As recently reported by Recchia et al., there were changes in 43 genes in each species, with 2 being common at an early time point (P13 in mice and P15 in rat) [39]. On the other hand, at a later time point (P18 in mice and P20 in rat), there were changes in 26 genes in the rat and 1622 in the mouse, with only 13 being common [39]. Although it is not clear how these gene changes affect retinal function, the differences in gene expression in both models may be partly involved in retinal function.

In addition to compromised ERGs, we observed significant decreases in the thicknesses of the IPL and INL in the OIR mice compared to those of normal mice with the recovery of a- and b-wave amplitudes (Figs. 1 and 3). Our finding that the reduction in a- and b-wave amplitudes does not correspond with structural degeneration concurs with the finding of a previous report [40]. The cellular origin of OPs is amacrine cells [41], which constitute 40% of all cells in the INL [42]. In addition, VEGF receptor activation results in the death of amacrine cell progenitors [43]. Therefore, thinning of both the INL and IPL may be related to retinal amacrine cell lesions in chronic OIR mice. The formation of capillary beds in the outer retina as far as the outer border of the INL occurs via budding from primary blood vessels [44]. Because there were lesions in both the INL and IPL, where retinal blood vessels are abundant, we examined in detail whether the OIR mice exhibited evident vascular pathology in the chronic phase. At 8 w, the OIR mice had no neovascular tufts but showed decrease in blood vessel length, area, branch points, and segments. These results indicate that decreased capillary density occurs in OIR mice. The decrease in capillary density might have an effect on retinal function, which is reflected in OP amplitudes. The murine OIR model is not identical to ROP in patients with respect to the angiogenic mechanisms, including vasoobliteration. However, the present study indicates that the murine OIR model is acceptable as an animal model because we investigated the interaction of the structural transition and retinal function in the chronic phase when the vasoobliteration had disappeared.

Next, to verify retinal neurovascular units in the chronic OIR mice, we evaluated both retinal permeability and expression of retinal microvascular tight junction molecules. We found that retinal hyperpermeability was observed with BRB disruption in chronic OIR mice, and we evaluated claudin-5 and occludin expression, which are involved in the assembly of tight junctions between neural microvascular endothelial cells [18], [20], [21], [28]. In the retinas from the chronic murine OIR model, the expression of both claudin-5 and occludin decreased. Because OPs are related to ocular pathology or abnormalities [45], [46], [47], OP attenuation in OIR mice might affect retinal hyperpermeability by decreasing both claudin-5 and occludin in the chronic phase. The present study is the first to show the importance of retinal permeability in retinal dysfunction. Recently, it was shown that OP-waves are markedly decreased in the region where retinal permeability was increased in patients with diabetes and clinically significant macular edema, as revealed by multifocal ERG [48]. The present data correlated with data of the previous report with respect to the relationship between OP-waves and retinal permeability.

In conclusion, these findings in the murine chronic OIR model indicate that deficits in OPs may be a sign of BRB disruption, which may be indicative of the early symptoms of retinal vascular abnormalities. The chronic OIR model revealed vascular impairment dysfunction without neovascular tufts; therefore, it may be appropriate to investigate the pathogenesis of ROP that leads to blindness caused by neovascularization without retinal detachment. The abnormalities of the OPs seen on ERG can be used to diagnose BRB disruption, and appropriate treatments, such as protection of endothelial cells in the early phase, may be able to prevent the loss of vision observed in ROP.

## Materials and Methods

### Animals

The experimental designs and all procedures were approved by the Animal Experimental Committee of Gifu Pharmaceutical University (permission number; 2009-206, 2010-037). All experiments were performed in accordance with the ARVO Statement for the Use of Animals in Ophthalmic and Vision Research, and monitored by the Institutional Animal Care and Use Committee of Gifu Pharmaceutical University. C57BL/6 mice (SLC, Shizuoka, Japan) were used in this study. The mice were kept under controlled lighting conditions (12∶12-h light/dark).

### OIR model

The OIR mouse model was created as described previously [49]. In brief, P7 pups along with nursing mothers were placed in 75% oxygen. Pups were removed on P12, and oxygen was continuously monitored with an oxygen controller (PRO-OX 110; Reming Bioinstruments Co., Redfield, SD, USA). On P12, the mice were returned to room air until P17, 4 weeks (4 w), or 8 w. Control mice were reared in room air. For morphological analysis, eyes were enucleated at P17, 4 w, and 8 w in both normal and OIR model mice.

### Electroretinography

ERG measurements in mice were performed as described in our previous report [50]. Electroretinograms (ERGs) were recorded 5 days after light exposure (Mayo, Aichi, Japan). Mice were maintained in a completely dark room for 24 h before being, intraperitoneally anesthetized with a mixture of ketamine (120 mg/kg) (Daiichi-Sankyo, Tokyo, Japan) and xylazine (6 mg/kg) (Bayer Health Care, Tokyo, Japan). The pupils were dilated with 1% tropicamide and 2.5% phenylephrine (Santen, Osaka, Japan). Flash ERG was recorded in the left eyes of the dark-adapted mice by placing a golden-ring electrode (Mayo) in contact with the cornea and a reference electrode (Nihon Kohden, Tokyo, Japan) through the tongue. A neutral electrode (Nihon Kohden) was inserted subcutaneously near the tail. All procedures were performed in dim red light, and the mice were kept warm during the entire procedure; body temperature was maintained with a heating pad. All animals recovered from anesthesia after the ERG recording sessions.

To demonstrate the oscillatory potentials (OPs), the OP amplitudes were measured in the time between the a- and b-wave peaks; the factors were OP number (OP2, OP3, OP4, and OP5), age (4, 6 and 8 w), and flash intensity [0.98 (log cds/m^2^)]. According to a previous report [51], the first OP wave is contaminated by the a-wave. Therefore, the first OP waves were not analyzed. The amplitude of the a-wave was measured from the baseline to the maximum a-wave peak, and the b-wave was measured from the maximum a-wave peak to the maximum b-wave peak according to age (4, 6, 8 w) and flash intensity [−2.92–0.98 (log cds/m^2^)]. In the present study, OPs amplitudes were measured by using ERG with all frequency (0.3–500 Hz).

### Histological analysis

In mice under anesthesia (80 mg/kg sodium pentobarbital, intraperitoneal), each eye was enucleated and kept immersed for at least 24 h at 4°C in a fixative solution containing 4% paraformaldehyde. Six paraffin-embedded sections (thickness, 5 µm) cut through the optic disc of each eye were prepared in a standard manner and stained with hematoxylin and eosin. The damage in OIR model mice was then evaluated with 3 sections from each eye used for the morphometric analysis as described below. Light microscope images were photographed using a digital camera (COOLPIX 4500; Nikon, Tokyo, Japan). The cells in GCL and the thicknesses of the IPL, INL, and ONL at a distance between 375 and 625 µm from the optic disc were measured on the photographs in a masked fashion by a single observer (H.O.). Data 3 sections (randomly selected from the 6 sections) were averaged for each eye and used to evaluate the cell count in the GCL and the thickness of the IPL, the INL, and the ONL.

### Imaging and quantification for retinal tube formation

Mice were sacrificed on P17 and perfused via the left ventricle with 1 mL of 20 mg/mL fluorescein isothiocianate (FITC)-dextran (Sigma–Aldrich, St. Louis, MO, USA) dissolved in PBS. Eyes were enucleated and fixed for 1–6 h in 4% paraformaldehyde, and retinal flat-mounts were prepared. Total images of flat-mounted retinas were produced via Metamorph (Universal Imaging Corp., Downingtown, PA, USA). To evaluate the retinal vascular lumen, we used the Angiogenesis Tube Formation module. Tube length, area, branch points, and segments were quantified from these images. We evaluated the number of nodes and the node area, which is the region containing connected blobs with a thickness exceeding the maximum width of the vessels; this represents an area where there is pooling of fluorescein-conjugated dextran.

### Retinal permeability

The permeability of mouse retinal vessels was determined as described previously [20], [28]. Briefly, under deep anesthesia with pentobarbital sodium, the mouse chest cavity was opened and was inserted into the left ventricle. Each mouse was perfused with 1 mL of PBS containing 100 µg/ml Hoechst 33342 (molecular mass, 616 Da; Sigma) and 20 mg/ml FITC-dextran (molecular mass, 2000 kDa). The isolated retinas were flat mounted and observed by a confocal microscopy (FluoView FV10; Olympus, Tokyo, Japan). Moreover, to quantify retinal permeability using Metamorph, fluorescent images were photographed (200×, 0.144 mm^2^) using an epifluorescence microscope (BX50; Olympus) fitted with a CCD camera (DP30VW; Olympus). Hoechst 33342 that did not merge with FITC-dextran at a distance of 1 mm from the optic disc were evaluated on the photographs in a masked fashion by a single observer (S.N.). Data from the 4 parts of each eye were used (total area, 0.576 mm^2^). The following equation was used to calculate retinal permeability rate:
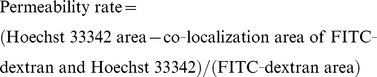



### Western blot analysis

*In vivo*, both normal mice and OIR model mice were euthanized at 8 w using 80 mg/kg sodium pentobarbital intraperitoneally, and their eyeballs were removed quickly. The retinas were carefully separated from the eyeballs and quickly frozen in dry ice. For protein extraction, the tissue was homogenized in cell lysis buffer using a Physcotron homogenizer (Microtec Co. Ltd., Chiba, Japan). The lysate was centrifuged at 12,000× *g* for 20 min, and the supernatant used for this study. The protein concentrations were measured by comparison with a known concentration of bovine serum albumin using a bicinchoninic acid protein assay kit (Pierce Chemical, Rockford, IL, USA). A mixture of equal parts of an aliquot of protein and sample buffer with 10% 2-mercaptoethanol was subjected to 5%–20% sodium dodecyl sulfate-polyacrylamide gel electrophoresis. The separated protein was then transferred onto a polyvinylidene difluoride membrane (Immobilon-P; Millipore Billerica, MA, USA). For immunoblotting, the following primary antibodies were used: claudin-5 mouse monoclonal antibody (Invitrogen, CA, USA) (1∶1000), occludin rabbit polyclonal antibody (Santa Cruz Biotechnology, CA, USA) (1∶200) and β-actin mouse monoclonal antibody (Sigma-Aldrich) (1∶4000). Either goat anti-rabbit or anti-mouse horseradish peroxidase-conjugated (1∶2000) was used as a secondary antibody. The immunoreactive bands were visualized using Super Signal® West Femto Maximum Sensitivity Substrate (Thermo Fisher Scientific K.K., Waltham, MA, USA), and measured using FLA-2000 (Fujifilm, Tokyo, Japan).

### Statistical analyses

Data are presented as the means ±standard error of the mean (S.E.M). The control mice participated in all experiments, and some OIR mice were used in multiple experiments. Statistical comparisons were made using Student's *t*-test [using StatView version 5.0 (SAS Institute, Cary, NC, USA)]. A *P* value of <0.05 was considered statistically.
